# XB130 translocation to microfilamentous structures mediates NNK-induced migration of human bronchial epithelial cells

**DOI:** 10.18632/oncotarget.3777

**Published:** 2015-04-20

**Authors:** Qifei Wu, Jeya Nadesalingam, Serisha Moodley, Xiaohui Bai, Mingyao Liu

**Affiliations:** ^1^ Latner Thoracic Surgery Research Laboratories, University Health Network, Toronto General Research Institute, Toronto, Ontario, Canada; ^2^ Department of Thoracic Surgery, the First Affiliated Hospital, School of Medicine, Xi'an Jiaotong University, Xi'an, China; ^3^ Institute of Medical Science, University of Toronto, Toronto, Ontario, Canada; ^4^ Department of Surgery, Faculty of Medicine, University of Toronto, Toronto, Ontario, Canada

**Keywords:** airway epithelial cell migration, F-actin association, cortactin, intracellular signal transduction

## Abstract

Cigarette smoking contributes to the pathogenesis of chronic obstructive pulmonary disease and lung cancer. Nicotine-derived nitrosamine ketone (NNK) is the most potent carcinogen among cigarette smoking components, and is known to enhance migration of cancer cells. However, the effect of NNK on normal human bronchial epithelial cells is not well studied. XB130 is a member of actin filament associated protein family and is involved in cell morphology changes, cytoskeletal rearrangement and outgrowth formation, as well as cell migration. We hypothesized that XB130 mediates NNK-induced migration of normal human bronchial epithelial cells. Our results showed that, after NNK stimulation, XB130 was translocated to the cell periphery and enriched in cell motility-associated structures, such as lamellipodia, in normal human bronchial epithelial BEAS2B cells. Moreover, overexpression of XB130 significantly enhanced NNK-induced migration, which requires both the N- and C-termini of XB130. Overexpression of XB130 enhanced NNK-induced protein tyrosine phosphorylation and promoted matrix metalloproteinase-14 translocation to cell motility-associated cellular structures after NNK stimulation. XB130-mediated NNK-induced cell migration may contribute to airway epithelial repair; however, it may also be involved in cigarette smoking-related chronic obstructive pulmonary disease and lung cancer.

## INTRODUCTION

Chronic obstructive pulmonary disease (COPD) affects over 300 million people worldwide, it was ranked as the third-leading cause of death, killing over 3 million people each year [1]. Lung cancer is the leading cause of cancer-related death throughout the world [2]. Cigarette smoking is the most common causes of COPD [3], and it is also a risk factor of lung cancer. Moreover, approximately 15% of all cancers worldwide are tobacco-related [4]. Nicotine-derived nitrosamine ketone (NNK), also known as 4-(methylnitrosamino)-1-(3-pyridyl)-1-butanone, is a nicotine-derived cigarette smoke component and a potent tobacco-specific carcinogen [4–6]. NNK accounts for approximately 27% of the end products formed in tobacco smoke, and it has been shown to be the most potent systemic lung carcinogen in rodents [7]. NNK can bind with high affinity to the cell surface protein, alpha-7 nicotinic acetylcholine receptor (α7nAChR) that is expressed in airway epithelial cells [4]. α7nAChR ligand-mediated activation results in the stimulation of several downstream pathways, including β-arrestin/Src, PI3K/Akt and noradrenaline/adrenaline/β-adrenergic receptor signalling-associated epidermal growth factor receptor (EGFR) pathways, which influence cell proliferation, survival, angiogenesis, epithelial to mesenchymal transition, and migration/invasion of cells [8]. NNK can enhance migration of lung, colon and gastric cancer cells [9–11]. Moreover, treatment of normal human airway epithelial cells with NNK promoted cell transformation by decreasing cellular dependence on extracellular growth factor stimulation, contact inhibition, and adherence to extracellular matrix [5, 10]. Understanding the effects and underlying mechanisms of NNK on normal bronchial epithelial cells is important for preventing COPD, lung cancer and other respiratory diseases.

Adaptor proteins act as docking sites for many effector molecules, thereby facilitating the transfer or relay of signals to other molecules and compartments within the cell. There are many different adaptor proteins in cells that are known to regulate different signal transduction pathways and different cellular functions, such as cell migration, invasion [12], and proliferation [13]. XB130 is a newly discovered adaptor protein and a member of actin filament associated protein family, also known as actin filament associated protein 1 like 2 (AFAP1L2) [14]. XB130 has three tyrosine containing SH2 domain binding motifs and one proline-rich SH3 domain binding motif located within its N-terminus, followed by two pleckstrin homology (PH) domains and a coiled-coil region in the C-terminus [15]. XB130 plays important role in signal transduction. It can interact with c-Src protein tyrosine kinase to mediate c-Src activation and down-stream AP-1 and SRE transactivation [14]. XB130 also interacts with the p85α regulatory subunit of PI3K [16], and through the PI3K/Akt pathway regulates cell survival, proliferation and gene expression [17–19].

Airways are constantly challenged with foreign materials and pathogens, and some insults result in airway epithelial injury [20]. Cell migration is an important process for normal development and in tissue repair. Thus, understanding how bronchial epithelial cellmigration and cell signaling pathways are regulated during smoke exposure may provide preventive and therapeutic strategies in cigarette smoking-related diseases. When cells were stimulated with either epidermal growth factor or protein kinase C activator, or transfected with constitutively active Rac, XB130 was shown to be involved in cytoskeletal rearrangement of lamellipodium, as well as in cell migration and invasion [21]. In the present study, we hypothesized that XB130 may be involved in the migration of human bronchial epithelial cells exposed to NNK.

## RESULTS

### NNK induces translocation of XB130 to actin filament enriched structures

**Figure 1:**
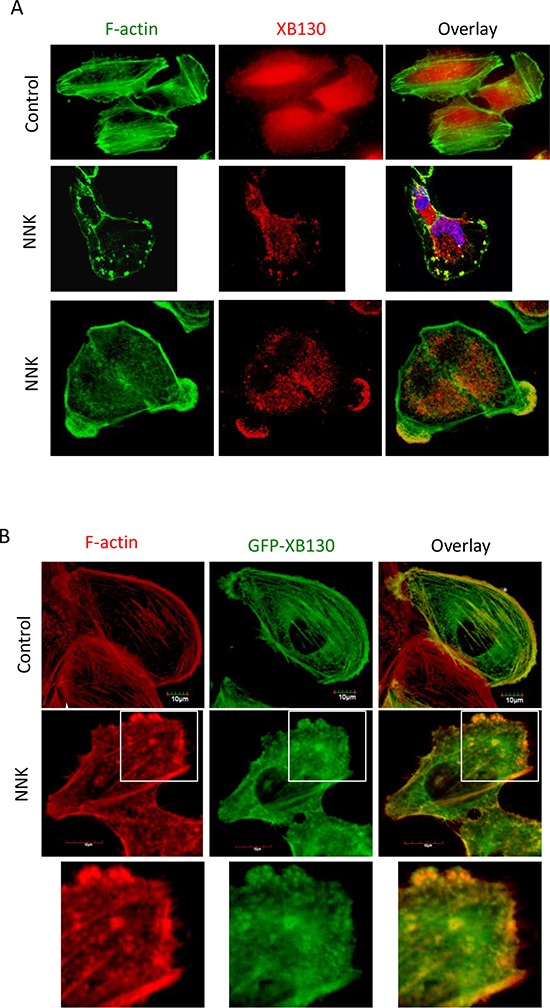
NNK-induces translocation of XB130 to actin-rich punctate and lamellipodia at the periphery of BEAS2B cells **A.** NNK-stimulation induced translocation of endogenous XB130 from cytoplasm to actin-rich small dots and lamellipodia at the periphery of the cells. BEAS2B cells were stimulated with or without 0.1 μM NNK for 30 min. Cells were immunostained with anti-human XB130 antibody and Alexa Fluor 594 labeled secondary antibody (red), and co-stained with Alexa Fluor 488 phalloidin (green). **B.** Overexpression of GFP-XB130 increased association of XB130 with actin stress fibers under control condition, and enhanced NNK-induced translocation of XB130 to actin-rich cellular structures. BEAS2B cells stably transfected with GFP-XB130 were treated with or with 0.1 μM NNK for 30 min. F-actin was stained with Alexa Fluor 594 phalloidin (red). Scale bar is 10 μm.

To determine the role of XB130 in the regulation of NNK-induced airway epithelial cell motility, normal human bronchial epithelial cells (BEAS2B) were stimulated with NNK. In BEAS2B cells, endogenous XB130 was mainly distributed in the cytoplasm, especially around the peri-nucleusregion (Figure 1A) [16]. However, in NNK stimulated cells, XB130 was re-distributed and enriched in cytoskeletal structures at the cell periphery. Co-staining for F-actin after NNK stimulation reveals that XB130 was enriched at punctate structures and in lamellipodia with F-actin (Figure 1A). We established BEAS2B cells stably expressing GFP tagged wild type XB130 (GFP-XB130). Similar as endogenous XB130, GFP-XB130 was also mainly distributed in the cytoplasm; however, it was also found along actin stress fibres and the cortical actin network (Figure 1B). This result suggests that even though XB130 does not have classical actin binding motif [22], it may be associated with filamentous actin structures, when its concentration is enhanced locally. Upon NNK stimulation, GFP-XB130 was redistributed and enriched in lamellipodia and ruffles along the cell's peripheral membrane and in small punctate in the cytoplasm, and again co-localizes with F-actin (Figure 1B).

### NNK-induced association between XB130 and actin cytoskeletal fraction

**Figure 2:**
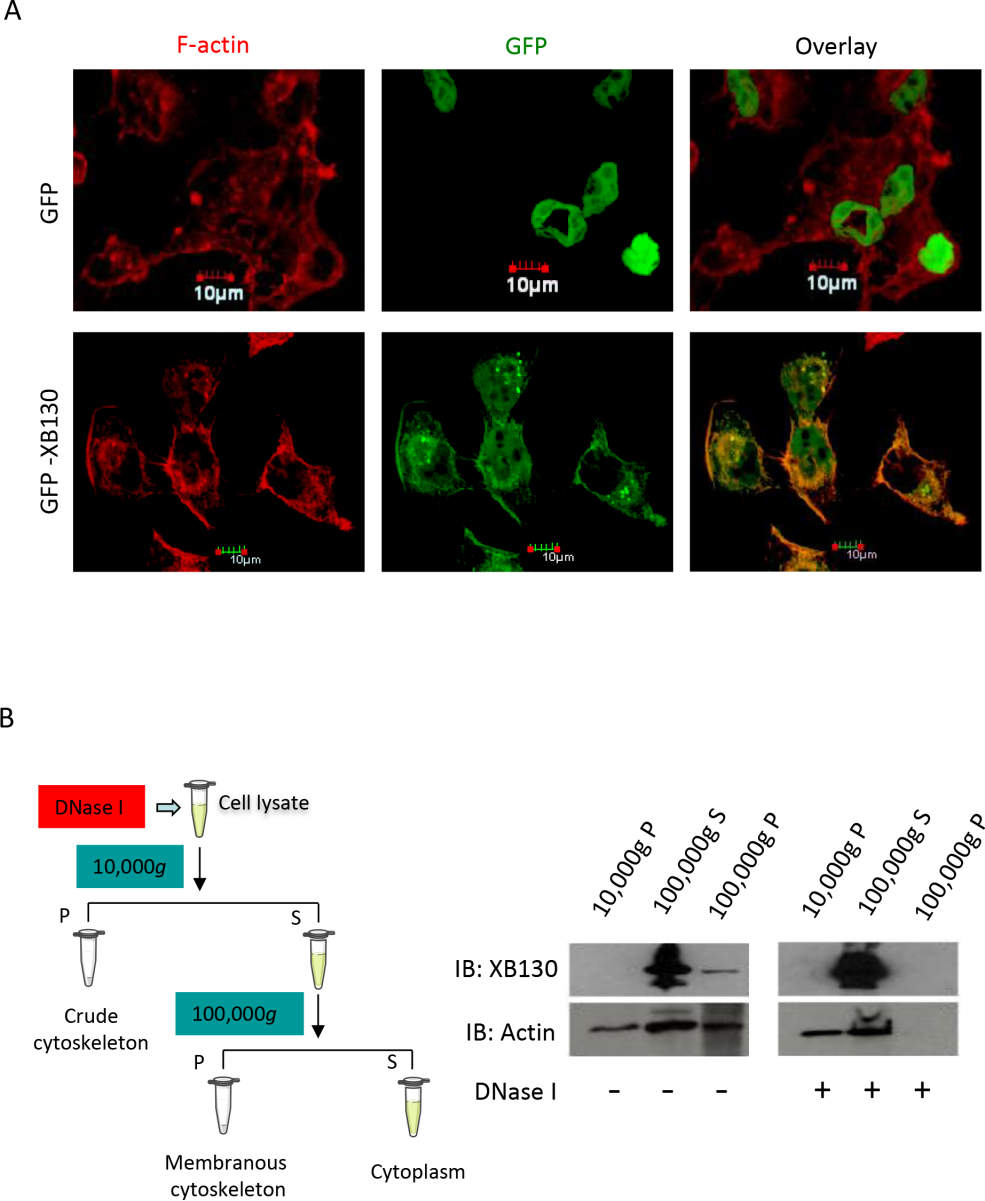
NNK induces association of XB130 with actin cytoskeletal structures. BEAS2B cells were stably transfected with GFP-XB130 or GFP alone **A.** GFP-XB130, but not GFP alone, is partially retained with actin cytoskeletal structures. After NNK stimulation (0.1 μM for 30 min), cells were treated with 0.2% Triton X-100 buffer for 5 min to remove cytoplasm. F-actin was stained with Alexa Fluor 594 phalloidin (red). Scale bar is 10 μm. **B.** XB130 is in the membranous cytoskeleton. GFP-XB130 cells were stimulated with 0.1 μM NNK for 30 min. Cell lysates were differentially centrifuged to collect the crude cytoskeleton fraction (10,000 *g*, pellet), cytoplasm fraction (100,000 *g*, supernatant), and membranous cytoskeleton fraction (100,000 *g*, pellet). XB130 was found mainly in the cytoplasm and membranous cytoskeletal fraction (see DNase I- group). DNase (0.5 mg/ml) treatment resulted in the loss of both actin and XB130 from the membrane cytoskeleton fraction of BEAS2B cell lysates (see DNase I+ group).

To further investigate XB130′s association with actin filaments, after NNK stimulation, we used 0.2% Triton X-100 buffer for 5 min to remove the cytosolic fraction of cells and looked for the presence of XB130 in the actin cytoskeletal fraction [23]. In cells expressing GFP alone, GFP signal in the cytoplasm was completely lost except in the nuclear region, and showed no association with F-actin staining (Figure 2A). However, in GFP-XB130 expressing cells, the GFP-XB130 fluorescence signal was reduced by detergent treatment but retained along the cell periphery and within diffusely distributed cytoskeletal structures (Figure 2A). These results show that after NNK stimulation, a fraction of XB130 is resistant to detergent extraction and associated with the cytoskeleton.

To further confirm that XB130 is associated with the membranous cytoskeleton, we isolated the cytoskeletal fraction from cell lysates using differential centrifugation. After high-speed centrifugation of cell lysates, highly cross-linked actin filaments, nucleus and cell debris can be pelleted. Ultra-speed centrifugation of the previously centrifuged supernatant allows further sedimentation of membranous actin filaments, which are often free or loosely cross-linked actin filaments. High- and ultra-speed centrifugation cytoskeletal extracts were prepared from NNK pre-treated GFP-XB130 expressing cells, and XB130 was found in the ultra-speed centrifugation pellet but predominantly found in the TritonX-100 soluble cytoplasmic fraction after ultra-speed centrifugation (Figure 2B, 100,000 *g*, S, DNase I-) and the XB130 in the crude cytoskeleton fraction was negligible (Figure 2B, 10,000 *g*, P, DNase I-). In contrast, actin was found in all three fractions.

To further determine whether XB130 was associated with actin, DNase I was added to the cell lysates to depolymerise filamentous actin [24] and to release associated proteins. The depolymerization of filamentous actin was confirmed by western blotting that shows the absence of actin after DNase I treatment (Figure 2B, 100,000 *g*, P, DNase I+). The depolymerization of F-actin also led to a partitioning of XB130 exclusively in the TritonX-100 soluble fraction (Figure 2B, 100,000 *g*, S, DNase I+). The above results further support that after NNK stimulation, XB130 is indeed associated with filamentous actin in membranous cytoskeleton.

### Overexpression of XB130 enhances NNK-induced cell migration

**Figure 3:**
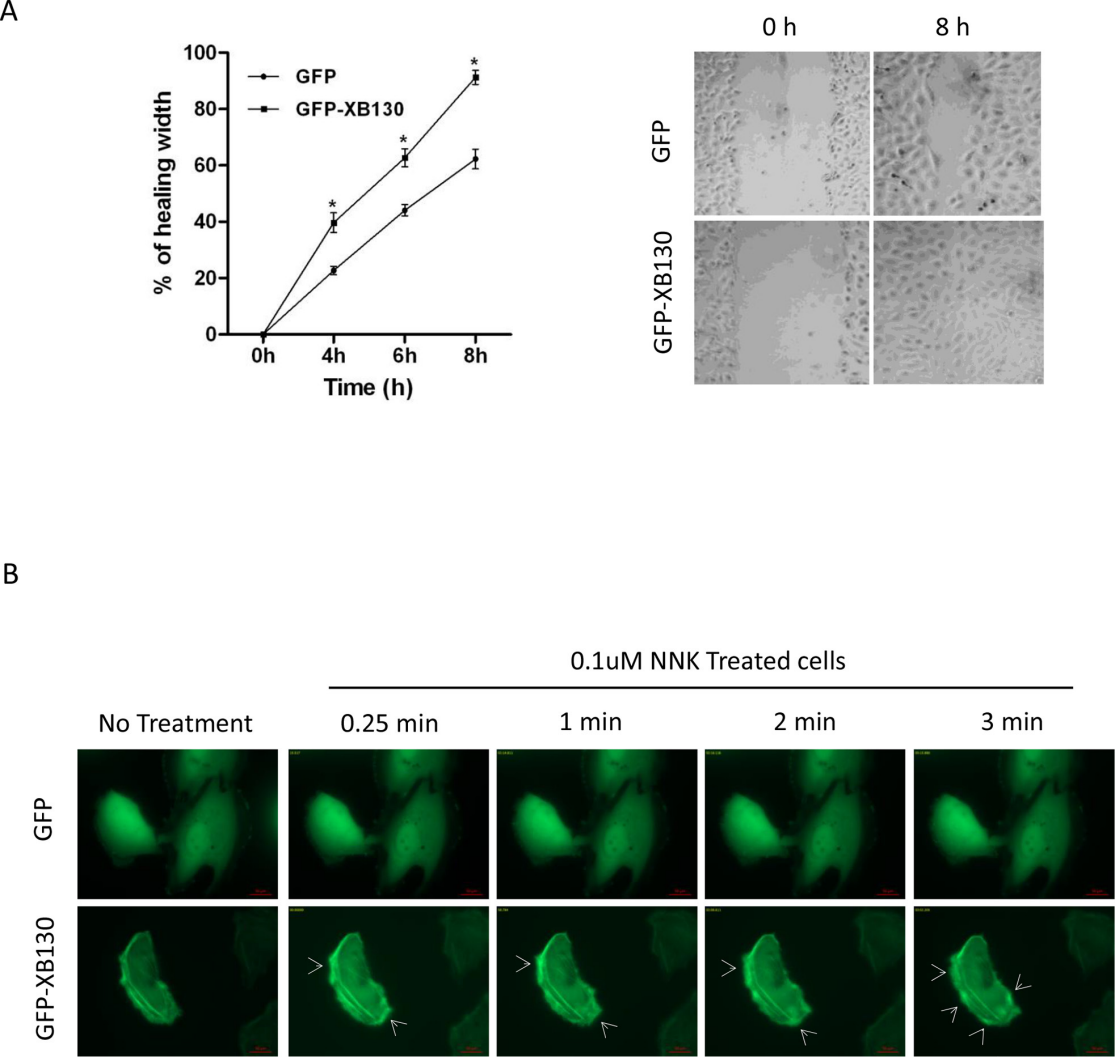
Overexpression of XB130 enhances BEAS2B cell migration. BEAS2B cells were stably transfected with GFP-XB130 or GFP alone **A.** Wound healing assays showed GFP-XB130 transfected cells showed increased cell migration as compared to GFP only transfected cells (*n* = 3, *P < 0.05). **B.** NNK-induced rapid translocation of XB130. BEAS2B cells stably transfected with GFP-XB130 or GFP alone were seeded in 4-well chamber slides. Cell images were captured before and after NNK treatment (0.1 μM for 15 min at 37°C, 5% CO_2_) using a Zeiss Apotome at 63x oil objective lens. Live cell-imaging shows that the distribution of GFP alone was not affected by NNK stimulation. By contrast, GFP-XB130 was seen at the front edge of the cell, which was further enhanced immediately after adding NNK (indicated by arrow head). GFP-XB130 also formed punctate structures inside of cells. Shown are representative images taken every minute after NNK treatment for a total of 3 min. Scale bar is 20 μm.

Our previous studies have shown that XB130 down-regulation in cells resulted in reduced cell migration in both wound healing assays and double chamber invasion assays [21]. In the present study, cells expressing GFP-XB130 showed markedly enhanced migration than cells expressing GFP alone in wound healing assays (Figure 3A). Eight hours after wounding of a confluent cell layer, the wounded area was about 95% closed in GFP-XB130 expressing cells, whereas, GFP alone expressing cells were only able to close the wound area by 60% (Figure 3A). Furthermore, live-cell imaging reveals that GFP-XB130 was enriched at the leading edge of the migrating cells, almost immediately after cells were stimulated with NNK (Figure 3B). These results support the notion that XB130 is involved in the formation of protrusions related to cell motility.

**Figure 4:**
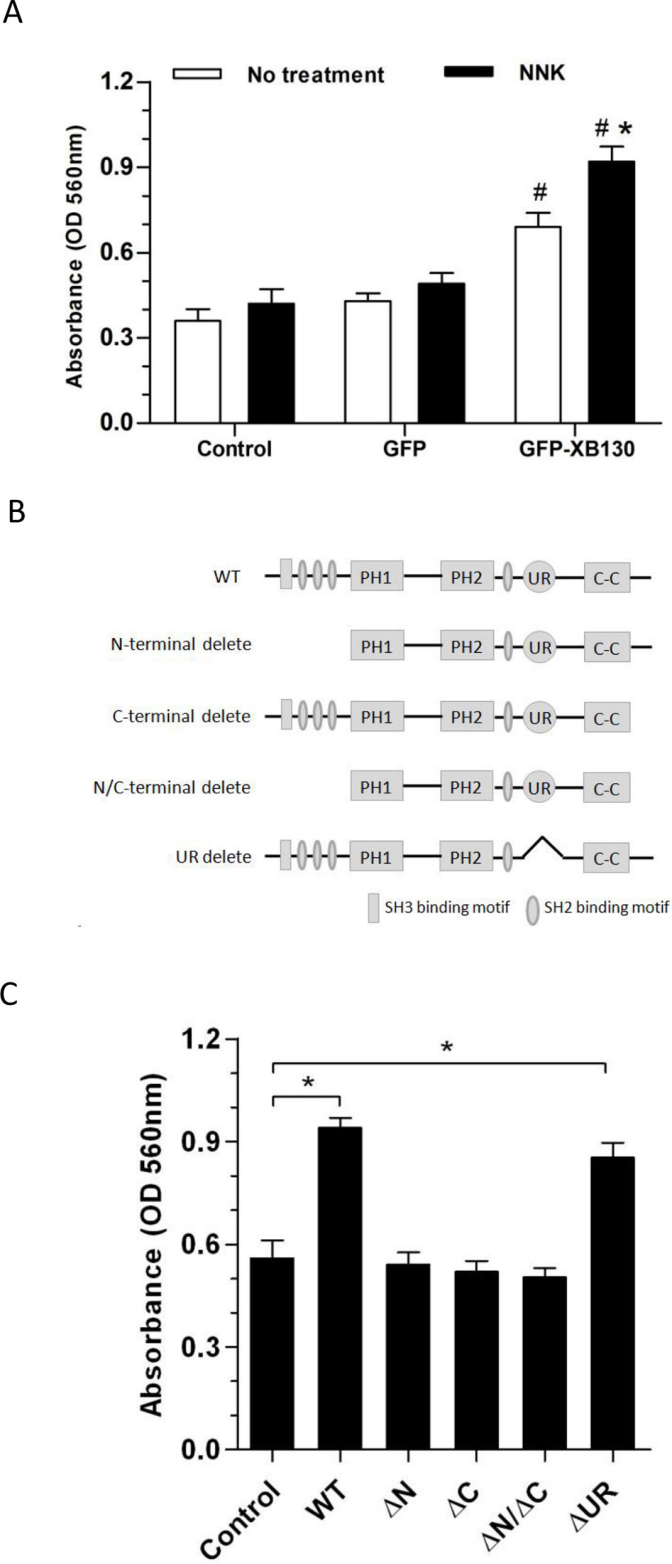
Overexpression of XB130 increased chemotactic cell migration **A.** GFP-XB130 enhanced NNK-induced cell migration. BEAS2B cells were seeded in QCM™ 24-well transwell chamber for chemotaxis cell migration assay. Cells were stimulated with 10% FBS together with or without 0.1 μM NNK for 16 h. Migration of GFP-XB130 transfected cells was significantly fast than that of GFP alone cells and Control cells (#: *P* < 0.05 *vs*. Control or GFP alone group). GFP-XB130 significantly increased NNK-induced cell migration (*n* = 3, *: *P* < 0.05, Compared between with and without NNK stimulation on GFP-XB130 groups). **B.** A schematic diagram representing the wild type full length XB130 protein and various deletion mutants used to develop stable transfection cell lines. **C.** The N- and C-termini of XB130 are required for mediating NNK-induced cell migration. Cells stably transfected with GFP-XB130 or its deletion mutants were stimulated with 10% FBS with 0.1 μM NNK for 16 h for chemotaxis cell migration assay. Deletion of N- and/or C-terminus of XB130 reduced NNK-induced cell migration (*n* = 3, *P < 0.05). UR: a unique region that is seen only in XB130, but not in other actin filament associate protein family members.

To determine whether XB130 can mediate NNK-induced cell migration, GFP-XB130 or GFP alone expressing BEAS2B cells were subjected to double chamber chemotaxis cell migration assay. GFP-XB130 expressing cells were about two-fold more migratory than GFP alone expressing cells or control BEAS2B cells. NNK treatment did not affect migration of control cells or cells expression GFP alone, by contrast, it significantly enhanced the migration of cells expressing GFP-XB130 (Figure 4A).

### Deletion of N- or C-terminus of XB130 decreased NNK-induced cell migration

We further sought to find out which domain(s) of XB130 is required for its participation in cell migration. We first established several XB130 deletion mutants (Figure 4B) tagged with GFP [16]. The GFP tagged XB130 mutants were made by 1) truncating the N-terminus region (aa 2–169) (GFP-XB130-ΔN), 2) truncating the C-terminus region (aa757–817) (GFP-XB130-ΔC), 3) truncating both N- and C-terminus regions (GFP-XB130-ΔC/ΔN), 4) selectively deleting a unique region (aa491–648) (GFP-XB130-ΔUR) that is presented in XB130, but not in other AFAP family members. BEAS2B cells stably expressing each of the above mutants were established.

We have found that the N-terminus and C-terminus of XB130 is responsible for translocation of XB130 to lamellipodia [21]. In agreement with the previous observations, GFP-XB130 significantly increased NNK-induced chemotaxis cell migration, which was significantly reduced in cells expressing N-terminus and/or C-terminus deleted mutants, but cell migration was not affected in cells with the unique region deleted mutant, as determined by the double chamber assay (Figure 4C).

### Deletion of N- or C- terminus of XB130 led to loss of its association with F-actin

**Figure 5:**
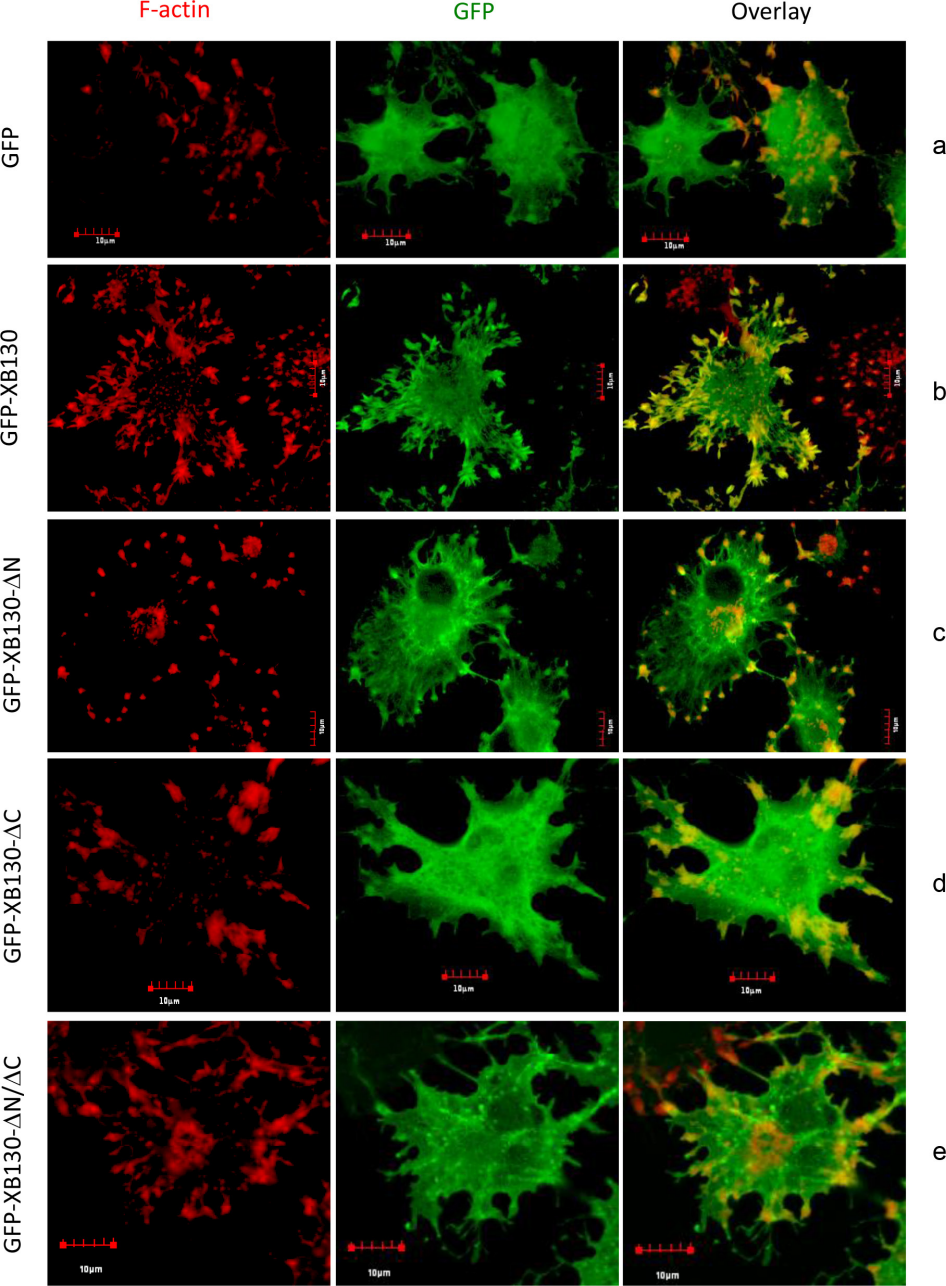
The C-terminus of XB130 is required for NNK-induced redistribution of XB130 to actin-rich aggregates Cells stably transfected with GFP-XB130 or its GFP-labeled mutants (green) were stimulated with 0.1 μM NNK for 30 min followed by 2 μM cytochalasin D treatment at 37°C for 1 h to inhibit F-actin polymerization. F-actin was stained with Texas-red conjugated phalloidin (red). Representative images show that deletion of the C-terminus or both C- and N-termini of XB130 prevented co-localization of GFP-XB130 with F-actin aggregates. Scale bar is 10 μm.

To investigate whether the reduced effects of XB130 mutants on NNK-induced cell migration is related to the association of XB130 with the actin cytoskeleton, BEAS2B cells stably expressing GFP alone, GFP-XB130, GFP-XB130-ΔN, GFP-XB130-ΔC, and GFP-XB130-ΔN/ΔC grown on coverslips were pre-treated with NNK for 30 min and then subjected to cytochalasin D treatment to block actin polymerization. After cytochalasin D treatment, F-actin stress fibers disappeared and F-actin appeared as aggregates along the cell periphery and in the cytoplasm (Figure 5, left column). These small aggregates may represent cell adhesions to the extracellular matrix. In GFP-XB130 expressing cells the distribution of XB130 was enriched and co-localized with those actin aggregates along the periphery of the cells (Figure 5B). In GFP-XB130-ΔN expressing cells, the enrichment of XB130 along the cell periphery was partially reduced (Figure 5C). In GFP-XB130-ΔC or GFP-XB130-ΔN/ΔC or GFP alone expressing cells, reorganization of F-actin into aggregates was similar to those of GFP-XB130 expressing cells, however, GFP-XB130-ΔC or GFP-XB130-ΔN/ΔC was not enriched along the cell periphery (Figure 5D–5E). In GFP alone expressing cells, the GFP signal was mainly located in the cytoplasm and no enrichment of GFP signal was detected along the cell periphery (Figure 5A).

### NNK-induced tyrosine phosphorylation of XB130

**Figure 6:**
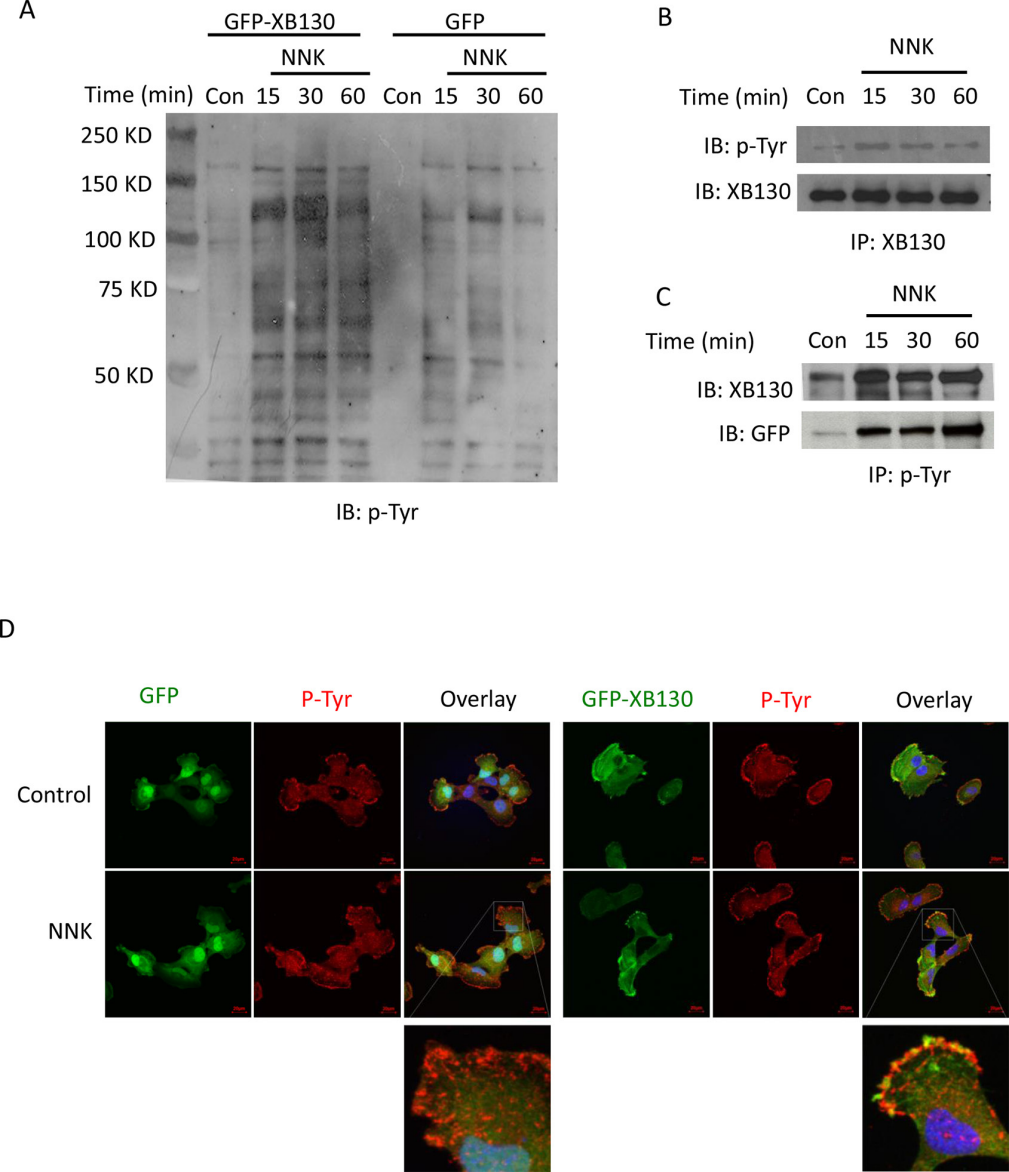
NNK treatment increased tyrosine phosphorylation of XB130 **A.** GFP-XB130 enhanced NNK-induced protein tyrosine phosphorylation. BEAS2B cells stably transfected with GFP-XB130 or GFP vector alone were stimulated with 0.1 μM NNK for up to 60 min. Immunoblotting with anti-phosphotyrosine antibody (p-Tyr) shows that NNK increased multiple protein tyrosine phosphorylation, which was further enhanced by XB130 overexpression. **B and C.** NNK enhanced XB130 tyrosine phosphorylation. GFP-XB130 cells were stimulated with 0.1 μM NNK for up to 30 min. Whole cell lysates were immunoprecipitated with anti-XB130 antibody **B.** or anti-phosphotyrosine antibody **C.** and immunoblotted with antibodies indicated. NNK enhanced XB130 tyrosine phosphorylation after 15 min. **D.** NNK-induced XB130 tyrosine phosphorylation at the lamellipodia. Cells stably transfected with GFP-XB130 or GFP vector alone were stimulated with 0.1 μM NNK for 30 min. Immunostaining for anti-phosphotyrosine antibody using Alexa Fluor 594 labeled secondary antibody (red) and detection of GFP signals show that NNK stimulation did not induced translation of GFP alone (top panel). By contrast, NNK increased protein tyrosine phosphorylation and co-localization of GFP-XB130 and phosphotyrosine at the cell periphery (see overlay and inset). Scale bar is 20 μm.

NNK is known to activate Src kinase [17]. XB130 contains a number of potential tyrosine phosphorylation sites and is known to interact with c-Src tyrosine kinase [14] and is also tyrosine phosphorylated by other protein tyrosine kinases [17]. In the present study, NNK stimulation increased tyrosine phosphorylation of multiple proteins in a time dependent manner. In GFP-XB130 expressing cells protein tyrosine phosphorylation was further enhanced (Figure 6A). In order to determine the tyrosine phosphorylation of XB130, cell lysates were immunoprecipitated with anti-XB130 antibody and blotted for anti-phosphotyrosine antibody. NNK increased tyrosine phosphorylation of GFP-XB130 (Figure 6B). When cell lysates were immunoprecipitated with anti-phosphotyrosine antibody and blotted for anti-XB130 or anti-GFP, increased tyrosine phosphorylation of XB130 was confirmed (Figure 6C). We immunostained cells with anti-phosphotyrosineantibody after NNK stimulation (0.1 μM, 30 min). GFP alone did not translocate to the cell periphery, however, co-localization of GFP-XB130 and anti-phosphotyrosine staining was observed at cellular protrusions (Figure 6D), suggesting that either XB130 itself is tyrosine phosphorylated or XB130 is associated with tyrosine phosphorylated proteins.

### XB130 associates with cortactin, a cortical actin binding protein

**Figure 7:**
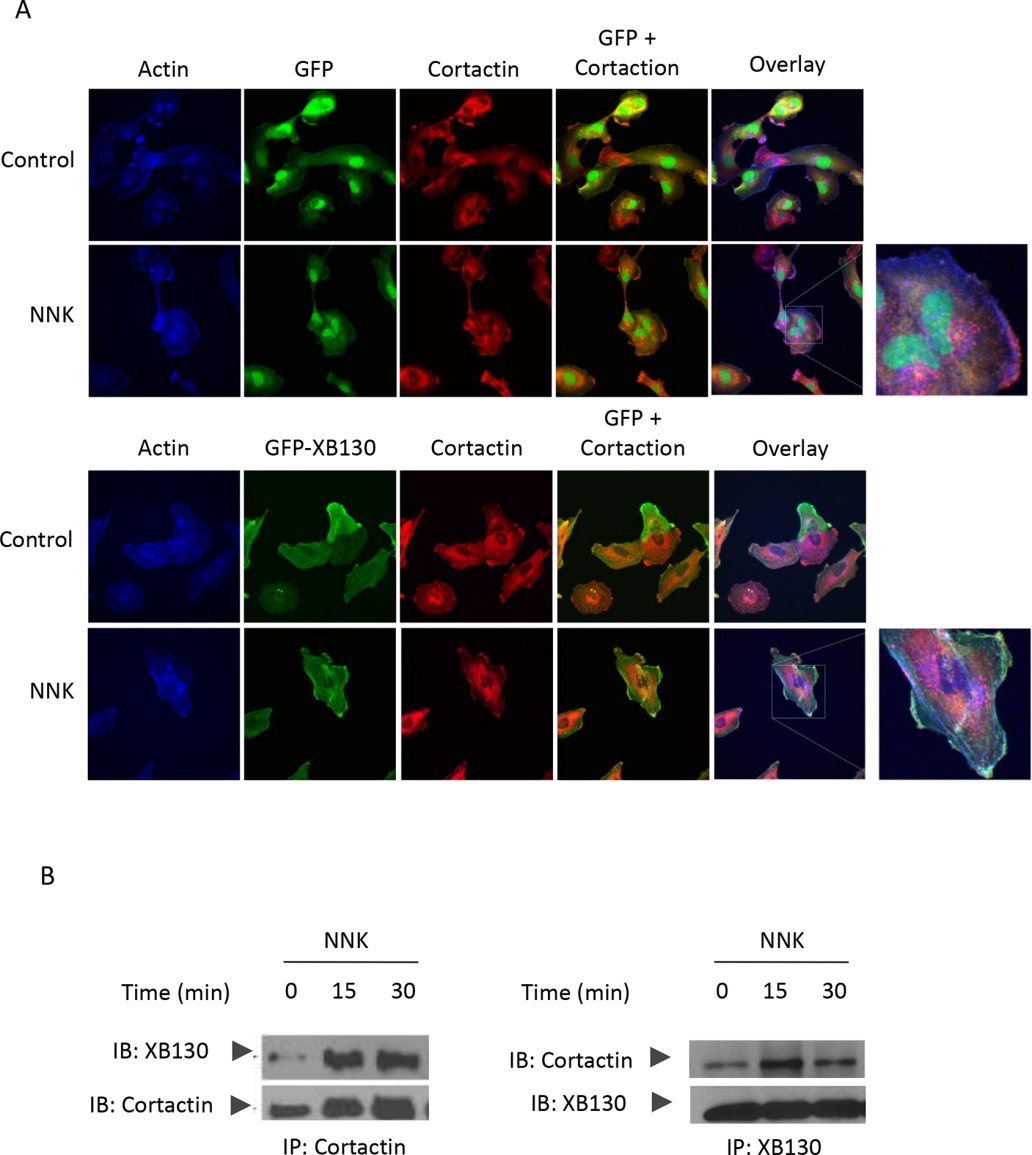
XB130/cortactin interaction is enhanced by NNK treatment in BEAS2B cells **A.** BEAS2B cells stably expressing GFP-XB130 or GFP alone were treated with 0.1 μM NNK for 30 min. Cells were stained with anti-cortactinantibody and Alexa Fluor 594 labeled secondary antibody (red), and Alexa Fluor 350 conjugated phalloidin (blue) for F-actin. In comparison with GFP alone transfected cells, GFP-XB130 enhanced NNK-induced translation of cortactin and colocalized with cortactin within actin-rich structures at the cell periphery. Scale bar is 50 μm. **B.** Co-immunoprecipitation between XB130 and cortactin was increased by NNK stimulation. GFP-XB130 stably transfected BEAS2B cells were treated with 0.1 μM NNK up to 30 min. Cell lysates were co-immunoprecipitated anti-cortactin or anti-XB130 antibody and immunoblots of XB130 and cortactin show that the interaction between XB130 and cortactin was enhanced by NNK stimulation.

Cortactin is a cortical actin binding protein in the cytoplasm that can be activated to promote polymerization and rearrangement of the actin cytoskeleton, especially the actin cortex around the cellular periphery [25, 26]. It plays an important role in promoting lamellipodial formation and cell migration [27]. To determine whether NNK stimulation can increase the interaction between XB130 and cortactin, we treated GFP-XB130 expressing cells with NNK for 30 min, followed by immunostaining for cortactin and fluorescent staining for F-actin. Under control condition, cortactin was located mainly in the cytoplasm. After NNK stimulation, cortactin and F-actin co-localization was found along the periphery of the cells; GFP-XB130, but not GFP alone was co-localized with cortactin as the F-actin enriched cellular protrusions (Figure 7A, as highlighted in the inset). Further, using GFP-XB130 expressing cells, we showed that anti-cortactin antibody pulled down XB130, and vice versa, anti-XB130 antibody also pulled down cortactin. The association between these proteins was enhanced by NNK treatment (Figure 7B).

### Overexpression of XB130 promotes matrix metalloproteinase-14 (MMP-14) translocation after NNK stimulation

**Figure 8:**
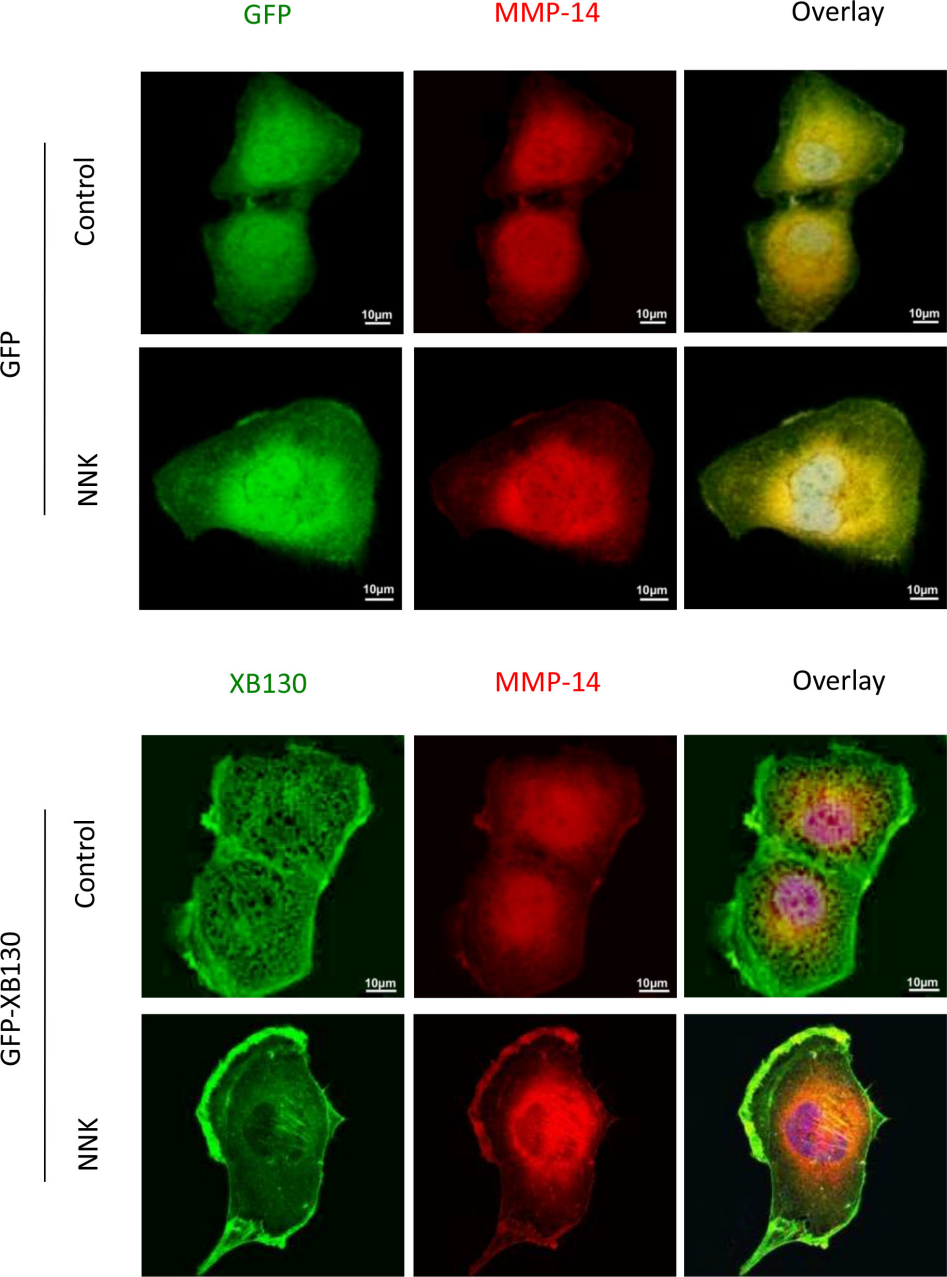
Over-expression of XB130 promoted MMP-14 translocation to actin-rich cellular structures BEAS2B cells stably transfected with GFP-XB130 or GFP alone were stimulated with 0.1 μM NNK for 30 min. Overexpression of XB130 enhanced NNK-induced MMP-14 (red) translocationto lamellipodia and ruffles on the cell peripheral membrane. Scale bar is 10 μm.

Matrix metalloproteinases (MMPs) are a family of proteinases capable of cleaving extracellular matrix proteins, they are sub-divided into soluble MMPs and membrane-type MMPs. MMP-14, a member of the membrane-type MMP subfamily, is the most important matrix metalloproteinase for cell migration and invasion [28] of human airway epithelial cells [29, 30]. Before NNK stimulation, MMP-14 was mainly found in the cytoplasm in both GFP-XB130 and GFP vector alone expressing cells (Figure 8). After NNK stimulation MMP-14 co-localized with GFP-XB130 but not with GFP alone, and was redistributed and enriched in lamellipodia and ruffles at the cell membrane (Figure 8). Similar to MMP-14, the expression of MMP-2 was also mainly localized in the cytoplasm before NNK stimulation, but after NNK stimulation the distribution of MMP-2 was not significantly changed (data not shown). Unlike MMP-14 or MMP-2, MMP-9 was enriched in focal adhesion-like cellular structures; however, this distribution pattern was not affected by NNK stimulation (data not shown).

## DISCUSSION

In this study, upon stimulation of human bronchial epithelial cells with NNK, cytoplasmic XB130 was translocated and enriched in cell motility-associated F-actin-rich cytoskeletal structures. Molecular structure-function studies demonstrate that both the N- and C-termini of XB130 are required for NNK-induced XB130 translation and XB130-enhanced cell migration. Furthermore, NNK treatment resulted in tyrosine phosphorylation of XB130 and interaction of XB130 with cortactin, which may be responsible for the translocation of XB130 to the cytoskeleton. XB130 also promoted MMP-14 translocation to cell motility related actin-rich cellular structures.

### NNK-induced association of XB130 with actin

Upon NNK stimulation, XB130 was relocated and enriched in lamellipodia and actin-rich small dots. Live cell-imaging reveals that XB130 was translocated to the leading edge of NNK-induced migrating cells. Immunostaining of cells after detergent extraction reveals that a distinct fraction of XB130 was retained with the F-actin cytoskeleton. Using cytoskeletal fractionation combined with actin depolymerisation, we show that the majority of XB130 was soluble in the cytoplasm, but a fraction of XB130 was pelleted with loosely cross-linked actin filaments and the membrane-bound cytoskeleton. Blocking actin polymerization followed by immunostaining and confocal imaging showed that XB130 was mainly associated with F-actin aggregates along the cell periphery, after NNK stimulation.

However, interaction between F-actin and XB130 was decreased upon deletion of either the N- or C-terminus of XB130. The N-terminus of XB130 contains several tyrosine phosphorylation sites, SH2 domain binding motifs and an SH3 domain-binding motif. Deleting these functional structures may reduce XB130 tyrosine phosphorylation and binding capacities with other F-actin-associated proteins. The C-terminus we deleted is only a few amino acids away from a coiled-coil region of XB130. The coiled-coil structure may be responsible for protein-protein interaction. C-terminus deletion may reduce the stability of XB130. The exact role of N- or C-terminus of XB130 in mediating its translocation to cytoskeletal structure needs to be further studied.

### Role of XB130 in NNK-induced tyrosine phosphorylation

Both endogenous XB130 and GFP-XB130 were tyrosine phosphorylated when cells were treated with NNK. On the other hand, overexpression of XB130 increased NNK-induced tyrosine phosphorylation of many proteins. Phosphorylation of proteins is a reversible enzymatic reaction that can switch “on” or “off” functions of proteins, as an important cellular regulatory mechanism [31]. Tyrosine phosphorylation of XB130, in response to NNK, may lead to conformational change of XB130 structure, and promote its association with other proteins, especially with proteins in specific cellular structures, such as lamellipodia. NNK is known to have high affinity to cell surface receptors, such as α_7_nAChR, in airway epithelial cells [17]. XB130 may mediate NNK-induced signal transduction downstream of α_7_nAChR in airway epithelial cells.

### NNK-induced association of XB130 with cortactin

Immunoprecipitation and immunostaining assays revealed that after NNK stimulation, XB130 was associated with another cytoplasmic protein, cortactin. Cortactin is known to bind to F-actin once it is phosphorylated by kinases [32]. Phosphorylated cortactin binds and recruits actin-related protein 2/3 complex, which is necessary to stimulate actin polymerization and branching in the fast-growing “barbed” end of existing F-actin cytoskeleton, especially in the actin cortex around the cell periphery [33]. Cortactin has a classical SH3 domain, which usually interacts with other cellular proteins by binding to proline-rich regions of the respective binding partners [34, 35]. XB130 has a proline-rich SH3 binding motif in the N-terminal region, which may mediate interaction between XB130 and cortactin. XB130 is a binding partner and an activator of Src kinase [14]. Cortactin is also a partner of Src kinase [36]. XB130 may enhance NNK-induced Src activation and cortactin tyrosine phosphorylation. Through interaction with cortactin, XB130 may promote actin cytoskeleton rearrangement and cell migration.

### XB130 and NNK-induced MMP-14 translocation

MMP-14 is involved in the remodelling of the ECM in physiological as well as pathological processes [37]. MMP-14 has emerged as a central regulator of proteolytic cell migration and invasion [38], which involves in the regulated turnover of ECM components and participates in the activation of secreted MMPs, such as pro-MMP-2 and pro-MMP-13 [39]. MMP-14 concentrates at the leading edge of various migrating cells after stimulation, and its focusing at specific sites of the cell surface is determined by interaction with other membrane proteins [37, 40]. In this study, overexpression of XB130 promotes MMP-14 translocation to actin-rich cellular structures after NNK stimulation. Recent studies show that vesicle-mediated transport of MMP-14 plays a key role in the regulation of surface-localised MMP-14 activity [41]. Regulators of vesicle formation, transport and fusion are thus expected to have a major impact on MMP-14 trafficking, and ultimately, on proteolytic cell migration and invasion [38]. XB130 may play a role in MMP-14 trafficking and facilitate cell migration after NNK stimulation.

In summary, NNK stimulation can enhance the migration of human normal bronchial epithelial cells and XB130 is a mediator for NNK-induced cell migration through its translocation to cell motility related microfilamentous cellular structures. This cellular process may be required for repair of airway epithelium damaged by cigarette smoking. However, extensive exposure to cigarette smoking may lead to airway remodeling and transformation of epithelial cells towards cancer cells. Whether XB130 is involved in the pathogenesis and progression of COPD and lung cancer merits further investigation.

## MATERIALS AND METHODS

### Reagents and antibodies

The following antibodies were used for analyses: monoclonal anti-actin (clone AC40) (Sigma-Aldrich Canada, Oakville, ON, Canada); anti-cortactin (p80/85, clone 4F11) and anti-phosphotyrosine (4G10) (Upstate biotechnology Inc., Lake Placid, NY, USA); anti-GAPDH and anti-GFP antibodies (clone SC40) (Santa Cruz Biotechnology, Santa Cruz, CA, USA); Anti-MMP-14, anti-MMP-2, and anti-MMP-9 antibodies (Biomol, Plymouth Meeting, PA, USA). The monoclonal XB130 antibody was generated in our lab as described previously [14]. The following secondary antibodies were used: peroxidase-conjugatedanti-mouse and anti-rabbit IgG were obtained from Amersham biosciences. AlexaFluor 350 phalloidin, Alexa Fluor 594 phalloidin and Texas-red conjugated phalloidin were purchased from Molecular Probes (Life Technologies, Rockville, MD, USA). NNK (Toronto Research Chemicals, North York, ON, Canada), paraformaldyhyde (Sigma-Aldrich Canada, Oakville, ON, Canada), Dulbecco's Modified Eagle's Medium (DMEM, Life Technologies, Rockville, MD, USA), fetal bovine serum (FBS, Gibco, Carlsbad, CA, USA), DMSO (Sigma-Aldrich Canada, Oakville, ON, Canada), natural polypropylene Eppendorf tubes for high speed centrifuge (Brinkmann Instrument ltd., Mississauga, ON, Canada); Neomycin (G418) and Fu Gene transfection reagent (Roche Diagnostics Canada, Laval, QC, Canada), penicillin and streptomycin (Life Technologies, Rockville, MD, USA), Dako fluorescence mounting medium (Dako Canada Inc., Mississauga, ON, Canada).

### Cell culture

Human airway epithelial cell line, BEAS2B, was purchased from ATCC (Manassas, VA, USA). Cells were cultured in DMEM (low glucose), with 10% FBS with penicillin (1mg/ml) and streptomycin (1 mg/ml), at 37°C and 5% CO_2_ humidified atmosphere following standard procedures.

### Immunofluorescence staining and confocal imaging

Cells cultured on cover slips were fixed with 4% paraformaldehyde in PBS for 20 min. Cells were permeabilized with 0.1% Triton X-100 for 20 min and incubated with 5% donkey goat serum in PBS for 1 h to block non-specific binding. Cells were incubated with primary antibody for 2 h or overnight and subsequently incubated with appropriate secondary antibody in PBS for 30 min. Cells were stained with phalloidin for 20 min to detect F-actin. All the steps were done at room temperature and cells were washed three times with PBS between each step. Cells were mounted on coverslips with Dako fluorescence mounting media. Images were captured using inverted laser scanning fluorescence confocal microscope (Olympus FluoView Confocal, FV1000-ASW, 60x oil objective) equipped with acquisition and analyses software.

### Transfection and stable cell selection

Plasmid constructs of GFP alone, GFP-XB130, GFP-XB130-ΔN, GFP-XB130-ΔC, GFP-XB130-ΔN/ΔC, and GFP-XB130-ΔUR, have been described [21]. BEAS2B cells cultured in 6-well plates were transfected with 1 μg of plasmids encoding the gene of interest, using 3 μL of Fu Gene6™ transfection reagent (Roche Diagnostics Canada, Laval, QC, Canada), according to the manufacturer's protocol. After 24 to 48 h incubation, transfection reagents were removed and replaced with new culture medium and maintained for 2 weeks. GFP positive cells were sorted into 96-well plates containing DMEM plus 30% FBS using MoFlo cell sorter (Dako Cytomation, Carpinteria, CA, USA) and expanded.

### Filamentous actin structurestaining

Cells cultured on glass coverslips were treated with 0.1 μM NNK at 37°C for 30 min., and washed twice with PBS. Then the cells were incubated in PBS containing 4 μmol/L glycerol, 25 mmol/L PIPES, 1 mmol/L EGTA, 1 mmol/L MgCl_2_, and 0.2% Triton X-100, pH 6.9, at room temperature for 5 min, then stained with phalloidin for 20 min to detect F-actin.

### Ultra-speed centrifugal preparation of cytoskeleton fractions

Cytoskeletal fractions of the cells were prepared with ultra-speed centrifugation [42]. In brief, cells were grown to about 90% confluence, treated with 0.1 μM NNK at 37°C for 30 min, washed with cold PBS and lysed with pre-cooled lysis buffer which contains 100 mmol/L Tris-HCL buffer (pH 7.6, 100 mmol/L NaCl, 10 mmol/L EGTA, 1 mmol/L MgCl_2_, 1 mmol/L PMSF, 10 mmol/L NaF, 2 mmol/L Na_3_VO_4_, 10 mmol/L β-glycerophosphate, 0.2 mmol/L ATP, 2% Triton X-100, 5 μg/ml aprotinin, and 5 μg/ml leupeptin) with or without DNaseI (0.5 mg/ml) on ice. After 15 min incubation at 4°C on a shaker, the lysates were centrifuged at 10,000*g* at 4°C for 10 min. The supernatant was further centrifuged at 100,000*g* at 4°C for 3 h to obtain soluble fraction and the membrane cytoskeleton in the pellet. The pellet was washed three times with lysis buffer and resuspended in 100 μl lysis buffer. Equal volumes of 10,000*g* crude cytoskeleton fraction (10,000*g*, pellet), 100,000*g* membranous skeleton fraction (100,000*g*, pellet), and Triton-X soluble fraction (100,000*g*, supernatant) were resolved by SDS-PAGE and blotted with antibodies against XB130 and actin.

### Western blotting

Cells were lysed using RIPA buffer (pH 7.5, 50 mmol/L Tris-HCl, 1 mmol/L EDTA, 100 mmol/L NaCl, 1% Triton X-100) containing protease inhibitors (20 μmol/L leupeptin, 0.8 μmol/L aprotinin, 10 μmol/L pepstatin and 1.25 mmol/L phenylmethylsulfonyl fluoride). Cell lysates were incubated on ice for 15 min and centrifuged at 14, 000*g* for 10 min at 4°C. Total protein in the supernatant was quantified using BCA protein assay reagent (Bio-Rad, Mississauga, ON, Canada), and equal amount proteins were loaded for SDS-PAGE. For western blots, proteins were transferred from gels to nitrocellulose membranes using a Miniprotein III electro blotter (Bio-Rad, Mississauga, ON, Canada). Immunoblots were washed in PBS containing 0.1% Tween-20 and then probed overnight at 4°C with primary antibody. Membranes were washed and incubated for 30 min at 4°C with the secondary antibody. Bound antibodies were detected using enhanced luminol and oxidizing reagents (ECL, Amersham Pharmacia Biotech, Freiburg, Germany).

### Time-lapse microscopy of live cells

BEAS-2B cells were seeded onto LabTek 4 well chamber slides and incubated at 37°C, 5% CO_2_ for 18 h in normal DMEM culture media. Cells were washed with PBS and incubated at 37°C, 5% CO_2_ for 1 h in phenol red free DMEM culture media supplemented with 10% FBS and 1 mg/mL penicillin and streptomycin. In an environment chamber attached to a Zeiss Apotome (Carl Zeiss, Oberkochen, Germany), live cell were imaged using a 63x oil objective lens for 1 min. at four different locations. The X, Y and Z coordinates at each location were set in the Zeiss Zen Prosoftware. Cells were treated with 0.1 μM NNK at 37^°^C, 5% CO_2_ for 15 min. Images were captured every minute at each location. Images were analysed using Zeiss Zen Pro.

### Wound healing assay

Cell migration was assessed with wound healing assays as previously described [43]. BEAS2B cells stably expressing GFP alone or GFP-XB130 plated on coverslips were cultured in 6-well tissue culture dishes. Confluent cell layers were manually scratched with a micropipette tip. Wells were rinsed once with PBS, replaced with new DMEM containing 10% FBS and incubated at 37°C and 5% CO_2_ in a humidified incubator. Images were captured at 0, 4, 6, and 8 h after wounding using Nikon Eclipse TE300 microscope. The wound distances from the images were measured using Image J program. The ratio of the final wound width to the width immediately after scratching is indicated as percentage of closure.

### Transwell cell migration assay

Cell migration was assayed using a QCM™ 24-well colorimetric cell migration assay kit (Millipore Corporation, Billerica, MA, USA) following the manufacturer's instructions. Cells were stimulated with 0.1 μM NNK in 10% FBS added to the lower chamber. After 16 h, non-migratedcells on the upper side of the filter were removed with a cotton swab; cells on the underside of the filter were stained with a cell stain solution, then subsequently extracted and detected on a standard microplate reader (at 560 nm wavelength).

### Blocking actin polymerization

To determine the association between XB130 and F-actin aggregates, an actin polymerization blocking strategy was used [44]. Briefly, cells were grown on glass coverslips and treated with 0.1 μM NNK for 30 min. NNK treated cells were then treated with 2 μmol/L cytochalasin D, an actin polymerization blocking reagent, in culture medium at 37°C for 1 h and then stained with phalloidin for 20 min to detect F-actin.

### Immunoprecipitation

Cell lysates were prepared with 0.5 ml of RIPA buffer per 100 mm culture dish. Cell lysates were diluted in NET buffer (50 mmol/LTris-HCl, pH 7.4, 150 mmol/L NaCl, 5 mmol/L EDTA, 0.05% Nonidet P-40) and incubated overnight at 4°C with 2 μg of anti-XB130, anti-phosphotyrosine, or anti-cortactin antibody. Three μg of protein G-sepharose beads (Amersham Pharmacia Biotech, Piscataway, NJ, USA) were added to the cell lysate-antibody mix and incubated at 4°C for 1 h. Bead-bound complexes were washed three times with cold NET buffer and denatured in Laemmli buffer at 100°C for 5 min. Samples were analysed by western blotting.

### Statistical analysis

Statistical significance was determined by Student's *t*-test or one-way ANOVA as appropriate, using GraphPad Prism 5.0 software. The significance cut-off was set to *P* < 0.05. The values are denoted asmean ± S.D.
